# Dopamine Regulates Approach-Avoidance in Human Sensation-Seeking

**DOI:** 10.1093/ijnp/pyv041

**Published:** 2015-04-09

**Authors:** Agnes Norbury, Zeb Kurth-Nelson, Joel S. Winston, Jonathan P. Roiser, Masud Husain

**Affiliations:** Institute of Cognitive Neuroscience (Ms Norbury and Drs Winston and Roiser), Wellcome Trust Centre for Neuroimaging (Drs Kurth-Nelson and Winston), Max Plank-UCL Centre for Computational Psychiatry and Ageing (Dr Kurth-Nelson), University College London, London, United Kingdom; Department of Experimental Psychology and Nuffield Department of Clinical Neurosciences, University of Oxford, Oxford, United Kingdom (Professor Husain).

**Keywords:** sensation-seeking, impulsivity, dopamine, D2 antagonist, addiction

## Abstract

**Background:**

Sensation-seeking is a trait that constitutes an important vulnerability factor for a variety of psychopathologies with high social cost. However, little is understood either about the mechanisms underlying motivation for intense sensory experiences or their neuropharmacological modulation in humans.

**Methods:**

Here, we first evaluate a novel paradigm to investigate sensation-seeking in humans. This test probes the extent to which participants choose either to avoid or self-administer an intense tactile stimulus (mild electric stimulation) orthogonal to performance on a simple economic decision-making task. Next we investigate in a different set of participants whether this behavior is sensitive to manipulation of dopamine D2 receptors using a within-subjects, placebo-controlled, double-blind design.

**Results:**

In both samples, individuals with higher self-reported sensation-seeking chose a greater proportion of mild electric stimulation-associated stimuli, even when this involved sacrifice of monetary gain. Computational modelling analysis determined that people who assigned an additional positive economic value to mild electric stimulation-associated stimuli exhibited speeding of responses when choosing these stimuli. In contrast, those who assigned a negative value exhibited slowed responses. These findings are consistent with involvement of low-level, approach-avoidance processes. Furthermore, the D2 antagonist haloperidol selectively decreased the additional economic value assigned to mild electric stimulation-associated stimuli in individuals who showed approach reactions to these stimuli under normal conditions (behavioral high-sensation seekers).

**Conclusions:**

These findings provide the first direct evidence of sensation-seeking behavior being driven by an approach-avoidance–like mechanism, modulated by dopamine, in humans. They provide a framework for investigation of psychopathologies for which extreme sensation-seeking constitutes a vulnerability factor.

## Introduction

Sensation-seeking is a personality trait concerned with motivation for “intense, unusual and unpredictable” sensory experiences (Zuckerman, 1994) that constitutes an important and well-conceptualised individual difference (Roberti, 2004). Engagement in various sensation-seeking–type activities (eg, recreational drug consumption, risky driving and sexual behaviors) covaries across both adults and adolescents (Carmody et al., 1985; King et al., 2012). In addition, questionnaire-based measures of sensation-seeking personality have high heritability estimates (40–60%; Koopmans et al., 1995; Stoel et al., 2006) with rank order differences in scores remaining highly stable over time (Terracciano et al., 2011).

Extreme sensation-seeking has been implicated in a variety of psychopathologies with high social cost, including substance and gambling addictions (Zuckerman, 1994; Roberti, 2004; Perry et al., 2011). Among individuals with substance use disorders, higher sensation-seeking score is associated with earlier age of onset, increased polysubstance use, more severe functional impairment, and poorer overall treatment outcome (Ball et al., 1994; Staiger et al., 2007; Lackner et al., 2013). Identification of mechanisms underlying human sensation-seeking is therefore likely to have high clinical relevance.

Investigations of animal models of sensation-seeking have implicated variation in striatal dopamine function, particularly at D2 types (D2/D3/D4) dopamine receptors, in mediating individual preferences for novel or sensory stimulation-inducing choice options (Bardo et al., 1996; Blanchard et al., 2009; Shin et al., 2010). As the efficacy of striatal dopaminergic transmission is considered to be involved in the vigor of approach behaviors in response to salient stimuli (Ikemoto, 2007; Robbins and Everitt, 2007), one theoretical account proposes that the core basis for individual differences in sensation-seeking is in the differential activation of dopaminergic approach-withdrawal mechanisms in response to novel and intense stimuli (Zuckerman, 1990).

Consistent with this view, genetic and PET evidence has implicated differences in function at D2-type receptors in individual differences in human sensation-seeking (eg, Hamidovic et al., 2009; Gjedde et al., 2010). Crucially, however, lack of behavioral paradigms analogous to those in the preclinical literature has meant that it has not been possible to test the approach-avoidance hypothesis directly in humans. Such an approach has previously proved highly fruitful with respect to other facets of impulsivity (Winstanley, 2011; Jupp and Dalley, 2014).

Here, we first tested a novel instrumental task of human sensation-seeking–like behavior that involved the opportunity to self-administer mild (but nonpainful) electric stimulation (MES) during performance of an economic decision-making task. This task was designed to be analogous to a recent operant sensation-seeking paradigm developed for rodents (Olsen and Winder, 2009). We next used a within-subjects design to investigate the effects of the D2 dopamine receptor antagonist haloperidol on task performance in a different sample of healthy volunteers. We predicted that: (1) individuals high in trait sensation-seeking would assign a positive economic value to the opportunity to experience such an “intense and unusual” sensory stimulus; (2) this preference would be reflected in an approach-like speeded relative response time for these stimuli; and (3) such “behavioral sensation-seeking” would be disrupted by antagonism at D2 receptors, depending on baseline sensation-seeking performance (Norbury et al., 2013).

## Study 1

### Methods

#### Participants

Forty-five healthy participants (28 female), mean age 24.3 (SD 3.55), were recruited via internet advertisements (for further demographic information, see Table 1). This sample size was chosen to allow us to detect a moderate-strength relationship between task performance and self-reported sensation-seeking trait on the basis of previous findings that correlations between behavioral and questionnaire measures of other facets of impulsive behavior are modest in strength (correlation coefficients up to 0.40; eg, Helmers et al., 1995; Mitchell, 1999). An a priori power calculation determined that a sample size of 44 would be necessary to detect a correlation coefficient of 0.40 at a conventional power of 80% and alpha of 0.05. Exclusion criteria consisted of any current or past neurological or psychiatric illness, or head injury. All participants provided written informed consent and the study was approved by the University College London Ethics Committee.

**Table 1.** Demographic Information for Participants

	Study 1	Study 2
n (female)	45 (28)	28 (0)
Age (years)	24.3 (3.55)	22.3 (2.74)
Years of education	16.1 (3.1)	-
Raven’s 12-APM score	-	9.1 (2.5)
SSS-V-R total score (range)	261 (46) (162–352)	-
UPPS SS score (range)	-	23.2 (5.8) (18–47)
Alcohol (drinks per week)	3.7 (4.5)	5.9 (8.7)
Tobacco (cigarettes per week)	4.1 (10.2)	8.4 (18.3)
Other drug use (n)		
None	30	18
Marijuana (ever)	8	5
Marijuana (regularly)	5	1
Stimulant use (ever)	2	4
Gambling behavior (n)		
Never	39	17
Several times per year	5	3
Several times per month	1	7
Weekly or more	0	1

Abbreviations: Raven’s 12-APM=Raven’s Advanced Progressive Matrices non-verbal IQ test (12-item version); SSS-V-R, Sensation-Seeking Scale version V (revised); UPPS SS, UPPS impulsivity scale sensation-seeking subscale score.

Other demographic scores refer to behavior during the last 12 months. Unless otherwise specified, figures represent mean (SD) for each group.

### Sensation-Seeking Task

**Figure 1.**
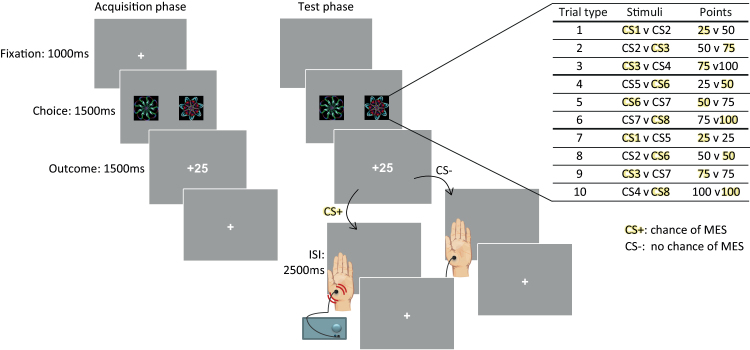
Sensation-seeking task. In the first part of the task (acquisition phase), participants were presented with a series of forced choice decisions between pairs of abstract fractal images. There were 8 different fractal stimuli (conditioned stimuli [CSs]) with 2 different CSs assigned to each of 4 possible point values (25, 50, 75, or 100 points; with which choice option a particular fractal represented randomised for every participant). Choice pairs were restricted to consist of either adjacent or equal points value stimuli, yielding 10 trial types. The acquisition phase of the task continued for a minimum of 80 trials until participants reached a criterion level of performance, ≥80% higher points value choices over the last 10 trials where a higher points value choice was possible. After this learning stage was completed, participants progressed to the second part of the task (test phase). For the test phase, participants were instructed that all stimuli were associated with the same points value as before but that some of the stimuli were now associated with the chance of receiving a mild electric stimulus (MES) to their nondominant hand (the magnitude of the MES was individually calibrated to be “stimulating but not painful” prior to starting the task). Specifically, one-half of the stimuli became designated as CS+s (chance of MES) and the other half CS-s (no chance of MES) in such a way that trials fell into 1 of 3 types: those where the CS+ was the lower points option, those where the CS+ was the higher points option, and, crucially, those where the CS+ and CS- stimuli were of equal points value. To increase the salience of the tactile stimulus, receipt of the electrical stimulation was probabilistic in both occurrence and timing. The probability of receiving the MES given selection of a CS+ stimulus was 0.75, with the onset of the MES occurring randomly during a 2500-ms inter-stimulus interval (ISI), throughout which participants were presented with a blank screen.

Participants completed a novel sensation-seeking task designed to probe the precise economic value (positive or negative) they assigned to the opportunity to receive an “intense” sensory stimulus (MES). In the first part of the task (acquisition phase), they simply learned the point values associated with various different abstract visual stimuli (conditioned stimuli [CSs]). Eight different fractals were used as CSs, with 2 of them assigned to each of 4 possible point values (25, 50, 75, or 100 points). In every trial, fractals were presented as pairs, restricted to consist of either adjacent or equal point value stimuli, yielding 10 different trial types (Figure 1).

The acquisition phase continued for a minimum of 80 trials until participants reached a criterion level of performance (choosing the fractal associated with the higher points value on 80% or greater of trials where this was possible, over the last ten trials). After this learning stage was completed, participants progressed to the second part of the task (test phase).

In the test phase, one-half of the choice stimuli became additionally associated with the chance of receiving a nonpainful MES to the hand. These fractals will henceforth be referred to as CS+s (for full details, see Figure 1). The other fractals were not associated with electrical stimulation and so are referred to as CS-. For each points value, one of the associated fractals became CS+ (chance of MES), while the other was CS- (no chance of MES). This yielded 3 trial types: those where CS+ was the lower points option, those where CS+ was the higher points option, and, crucially, those where the CS+ and CS- stimuli were of equal points value.

Participants thus continued to make choices between fractal pairs, with the only difference being that now one-half of the choice options were associated with the chance of receiving the MES, including, importantly, on trials where both fractals were of the same points value. The key experimental question was whether some participants’ choices would be biased towards selecting the CS+ stimuli when it was of equal points value to, or even less than, the CS-. The degree of bias in participants’ choice towards or away from CS+ stimuli, with respect to the relative points value of the CS+ option, thus allowed precise calculation of the economic value (positive or negative) each participant assigned to the opportunity to receive the additional intense sensory stimulus (see Computational modelling analysis).

Participants completed 100 test phase trials (10 per trial type) and were told they would be paid a cash bonus at the end that depended on the total number of points accrued. To increase the salience of the tactile stimulus, receipt of MES was probabilistic in both occurrence and timing. The probability of receiving the MES given selection of a CS+ stimulus was 0.75, with the onset of MES occurring randomly during a 2500-ms inter-stimulus interval.

Before initiating the task, participants rated their preference for each of the fractals to be used in the paradigm on a computerized visual analogue scale (VAS) (ranging from “like to “dislike”). This measure was repeated for a second time following completion of the acquisition phase (ie, after learning the points value associated with each CS), and for a third time at the end of the experiment (ie, following introduction of the MESs). For details of apparatus and stimulation parameters used to deliver the MES, see Supplementary Information.

### Design

Following consent and task instructions, the amplitude of the electrical stimulation was calibrated individually for each participant via a standardized work-up procedure. Specifically, participants received a series of single stimulation pulses, starting at a very low amplitude (0.5 mA; generally reported by participants as being only just detectable) and gradually increasing in current strength until the stimulation was rated as 6 out of 10 on a VAS ranging from 0 (just detectable) to 10 (painful or unpleasant), a level at which participants endorsed a description of the sensation as being “stimulating but not painful.” This procedure was repeated twice for each participant to ensure consistency.

Participants also completed several self-report measures: a revised measure of the Sensation-Seeking Scale version V (Zuckerman, 1994; Gray and Wilson, 2007); a measure of hedonic tone, the Snaith-Hamilton Anhedonia Scale (Snaith et al., 1995); and the trait scale of the State-Trait Anxiety Inventory (Spielberger et al., 1970). The latter 2 measures were included to test the possibility that individual differences in MES preference may be related to trait anxiety or current state (an)hedonia rather than being driven by sensation-seeking personality per se. Demographic information regarding years of education, cigarette and alcohol consumption, recreational drug use, and frequency of engagement in gambling-related activities was also collected.

### Computational Modelling Analysis

For test phase data, it was assumed that a choice between 2 CSs, A and B (where A is the CS+ stimuli and B is the CS-), could be represented as:

$${\text{V}}_{A}={\text{ R}}_{\text{A}}+\;\theta$$

$${\text{V}}_{B}={\text{ R}}_{\text{B}},$$

where R_X_ is the point value of stimulus X, θ is the additional value (in points) assigned to the opportunity to receive the MES (positive or negative), and V_X_ represents the overall value of each option.

This model was then fitted across all test phase choice data for each participant via a sigmoidal choice (softmax) function: 

$$\text{P}\left(\text{choose A}\right)\;=\text{ 1 }/\;\left(\text{1 }+\text{ exp}\left(-\beta *\left({\text{V}}_{A}-{\text{V}}_{B}\right)\right)\right)$$

Values of the free parameters θ and β (the softmax temperature parameter, a measure of choice stochasticity) were fitted to the data on a subject-by-subject basis using log likelihood maximization.

## Results

### Individual Differences in Preference for Additional Intense Sensory Stimulation

Overall, participants chose the MES-associated stimulus (CS+) on 20.4% (SD 17.6) of the trials where these represented the lower points option, 68.9% (24.8) of the trials where they were the higher points option, and 45.2% (19.9) of trials where CS+ and CS- stimuli were equal in points value. There was a significant effect of trial type on proportionate choice of CS+ stimuli (*F*_2,88_=157.29, *P*<0.001). Posthoc *t* tests revealed that overall participants chose the CS+ option significantly less frequently on lower point trials than equal point trials, and significantly more often on higher point trials than equal point trials (*t*_44_=-11.997, *P<*.001; *t*_44_=-8.102, *P<*.001, respectively).

**Figure 2.**
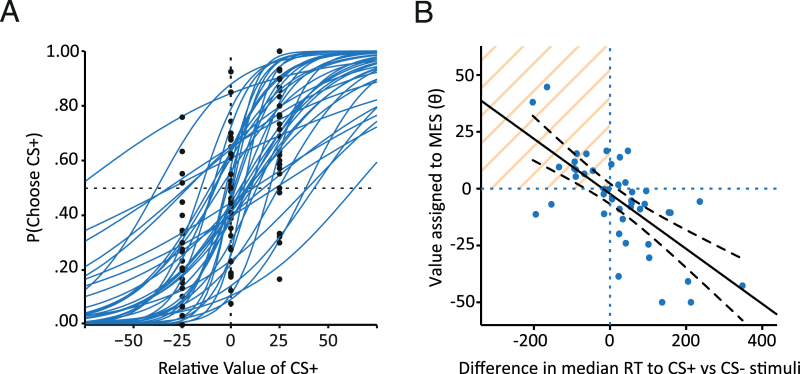
Interindividual variation in task performance. (*A*) Individual psychometric functions for probability of choosing the mild electric stimulation (MES; CS+ or MES-associated) option as a function of its relative points (monetary) value, generated for each participant from choice data across all trial types (black circles indicate actual proportionate choice for each trial type). The left/right translation of each function represents the influence of MES value (or θ) on choice, with the gradient of the function determined by the softmax temperature parameter β (a measure of the stochasticity of participants’ choice). A leftward shift in the function reflects a positive effect of opportunity for intense tactile stimulation on choice, that is, greater choice of the MES-associated options than would be expected from points-based choice alone. (*B*) The value an individual assigned to the opportunity to receive the MES (θ) strongly predicted their difference in choice reaction times (RTs) to CS+ vs CS- stimuli (median RT_CS+_ – median RT_CS-_; *r* =-0.690, *P<*.001). The opportunity for extra sensory stimulation slowed choice of these options in participants for whom it was aversive (low proportionate choice of the CS+; bottom right quadrant), but sped the choice in participants for whom it was appetitive (high choice of the CS+; top left quadrant, orange shading). Black dashed lines indicate 95% confidence intervals. n=45.

Importantly, there was substantial variation in preference for the MES-associated option on trials where CS+ and CS- options were equal in points value. Mean proportionate choice of CS+ stimuli ranged from 7.5% to 92.5% (Figure 2A; relative CS+ value of 0). An estimate of significantly biased choice on these trials can be made by sampling the binomial distribution; for 40 trials and an alpha of 0.05, this threshold is approximately 26/40 (0.65) for significantly high choice and 13/40 (0.35) for significantly low choice. Based on these thresholds, 8/45 (or 18%) of participants chose a significantly high proportion of CS+ stimuli, in other words, significantly sought the MES, and 13/45 (29%) of participants significantly avoided the CS+ options.

Consistently high choice of MES-associated stimuli was observed in a subset of participants even on trial types where the CS+ was the lower points value option, that is, involved sacrifice of economic value (Figure 2A, relative CS+ value of 25).

To test whether participants’ choice of the MES-associated stimuli varied significantly during the course of the task (ie, whether preference changed with decreasing stimulus novelty), test phase trials were binned into 4 sections. A repeated-measures ANOVA with the within-subjects factor of time (4 levels) found no evidence for a main effect of time-on-task on proportionate choice of CS+ stimuli across all subjects (*p*>.1). Overall choice of CS+ stimuli was also unrelated to number of trials taken to reach criterion performance or proportion of correct responses (higher point value choice on trials where this was possible) during the acquisition phase (*P*>.1), suggesting that preference for MES-associated stimuli was not associated with the learning of the points values during the first part of the task. MES preference was also not related to current amplitude (*P*>.1).

The computational modelling analysis describing the value (in points) that participants assigned to opportunity to receive the MES (θ) provided a good account of task performance (for details, see Supplementary Information). Figure 2B shows individual psychometric curves for probability of choosing the MES-associated option (CS+) as a function of its relative points (monetary) value, generated by fitting the model to choice data across all trial types for each participant.

### Relationship between Economic Value Assigned to Opportunity to Receive Intense Sensory Stimulation and Reaction Time for MES vs Non-MES–Associated Stimuli

Individual θ values were strongly negatively correlated with difference in choice reaction time (RT) for CS+ vs CS- stimuli (*r*=-0.690, *P<*.001) (Figure 2B). Specifically, participants who chose a greater proportion of MES-associated stimuli were faster to choose these stimuli (suggestive of conditioned approach). In contrast, participants who tended to avoid CS+ stimuli were slower to choose them (suggestive of conditioned suppression) (Pearce, 1997). This was not a time-on-task effect (eg, due to a tendency to decrease both mean RT and choice of the CS+ over the course of the task), as this relationship remained strongly significant when considering trials from only the latter half of the test phase (first half of trials *r*=-0.692, second half of trials *r*=-0.625, both *P<*.001).

### Relationship between Task Performance and Self-Report Measures

**Figure 3.**
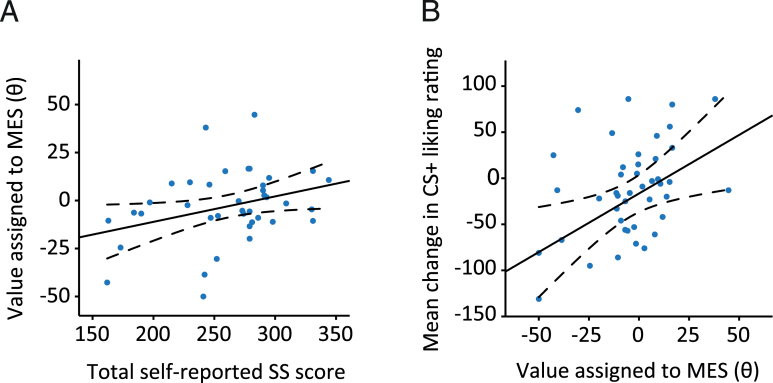
Relationship between task performance and self-report measures. (A) Total self-reported sensation-seeking score was significantly positively related to the value participants assigned to opportunity to receive mild electric stimulation (MES) (*r*=0.325, *P<*.05). (*B*) There was a positive relationship between value assigned to receipt of the intense sensory stimulation (θ) and mean change in visual analogue scale (VAS) “liking” rating of MES-associated (CS+) stimuli following the introduction of the additional electrical stimulation (*r*=0.368, *P<*.05). Dashed lines indicate 95% confidence intervals. n =45.

Individual θ values were significantly positively related to self-reported sensation-seeking score, such that participants who reported higher trait sensation-seeking assigned a greater value to opportunity to receive the MES (*r*=0.325, *P*=.043) (Figure 3A).

Theta value was unrelated to trait anxiety, self-reported hedonic tone, current amplitude, or years of education (all *P*>.1). Nonparametric tests were used to relate task performance to self-reported alcohol and tobacco use, as these data were substantially positively skewed. Independent-samples median tests revealed that individuals who assigned a positive value to the opportunity to receive the MES (ie, θ>0, n =17) smoked significantly more cigarettes per week (Fisher’s *P*=.006) and showed a nonsignificant trend towards consuming more alcoholic drinks per week (*P*=.098) than individuals who tended to avoid the MES (ie, θ<0, n=28) (mean cigarettes per week 6.7±10.4 vs 2.5±9.9; mean drinks per week 4.2±3.9 vs 3.4±4.9). There was no significant difference in mean θ value between individuals who did vs did not (n=15 vs n=30) report any recreational substance use other than alcohol or tobacco during the past 12 months (independent samples *t* test, *P*>.1) (Table 1). There was no difference in mean θ value between male and female participants (independent samples *t* test, *P*>.1).

MES value (θ) was also significantly positively related to mean change in VAS “liking” rating for CS+ stimuli following introduction of the MES (ie, between rating sessions 2 and 3; *r*=0.368, *P=*.013) (Figure 3B). Participants who assigned positive MES values tended to increase their liking rating of MES-associated stimuli, while participants with negative values tended to decrease their ratings.

Values of the model parameter indexing choice stochasticity (β; a measure of the extent to which participants’ choice was influenced by the difference in value between the 2 options) were unrelated to both self-reported sensation-seeking trait and θ values (*P*>.1), suggesting that higher sensation-seeking or MES-seeking individuals were not any less value-driven in their choice behavior than their lower sensation-seeking counterparts.

## Study 2

### Methods

#### Participants

Participants were 30 healthy males, mean age 22.3 (SD 2.74) (Table 1). Potential effects of haloperidol in female volunteers who might be pregnant precluded use of the drug in women in this study. Sample size (n=30) was based on the strength of the MES value/RT effect relationship we observed in Study 1. It was calculated that a sample of 29 participants should allow us to replicate (and detect any effects of haloperidol on) a true effect size of *r*=0.50 at a power of 80% and an alpha of 0.05. Exclusion criteria consisted of any current major illness, current or historic incident of psychiatric illness, and/or history of head injury. All subjects gave informed written consent and the study was approved by the University College London Ethics Committee.

### Design

The study was carried out according to a within-subjects, double-blind, placebo-controlled design. On the first session, participants gave informed consent and completed the sensation-seeking task in order to reduce the impact of any practice effects on performance across the subsequent 2 sessions (under placebo or drug). They then completed the UPPS impulsivity questionnaire (Whiteside and Lynam, 2001), which has subscales of sensation-seeking, and 3 other factor analysis-derived impulsivity facets. This measure was chosen to evaluate the selectivity of the relationship between task performance and sensation-seeking compared with other kinds of impulsivity. The sensation-seeking subscale of the UPPS is predominantly derived from items of the SSS-V, and therefore scores on the 2 measures intercorrelate highly (Whiteside and Lynam, 2001). A standardized, nonverbal measure of mental ability was also administered (Raven’s 12-item Advanced Progressive Matrices; Pearson Education, 2010).

On the second and third sessions, participants arrived in the morning and were administered either 2.5mg haloperidol or a placebo (drug and placebo were indistinguishable). A dose of 2.5mg haloperidol was chosen in order to be greater than that given in a previous study where inconsistent drug effects were observed (2mg; Frank and O’Reilly, 2006), but less than that used in other behavioral studies where significant negative effects of haloperidol on mood or affect were detected (3mg; Zack and Poulos, 2007; Liem-Moolenaar et al., 2010). Testing commenced 2.5 hours after ingestion of the tablet in order to allow drug plasma levels to reach maximum concentration (Midha et al., 1989; Nordström et al., 1992).

Following this uptake period, participants completed VAS measures of mood, affect, potential physical side effects, and knowledge of the drug/placebo manipulation. The Addiction Research Centre Inventory of psychoactive drug effects (ARCI; Martin et al., 1971) was also administered, as this previously has been shown to be sensitive to haloperidol (Ramaekers et al., 1999). Participants further completed 1 of 2 equivalent forms of the letter-digit substitution test (LDST; van der Elst et al., 2006), a simple pencil-and-paper test of general psychomotor and cognitive performance. Arterial heart rate and blood pressure were monitored pre- and post-drug administration.

The sensation-seeking task was as described for Study 1. For this study, participants completed an additional set of VAS ratings at the end of the task to test learning of CS+/CS- (MES-associated vs non-MES–associated) contingencies. For each CS, participants rated how strongly they believed choosing that stimulus had been associated with the chance of receiving electrical stimulation (“no chance of shock” to “chance of shock”). The individualized work-up procedure was repeated on every session to ensure that subjective intensity (as opposed to actual current amplitude) was matched across sessions. Drug/placebo order was counterbalanced across subjects, with a minimum of a 1-week washout period between the 2 test sessions (the mean time between visits was 18 days).

### Analysis

Computational modelling analysis of the sensation-seeking task was as described for Study 1. A repeated-measures ANOVA with the within-subjects factor of drug (haloperidol vs placebo), and the between-subjects factor of drug order (first vs second test session) was used to analyze key dependent variables from test session data. Specifically, these were participant-determined current amplitude, modelling parameters describing MES value (θ) and choice stochasticity (β), mean choice RT, and individual RT effect (median RT_CS+_ – median RT_CS-_). All reported simple effects analyses are via pairwise comparison with the Bonferroni adjustment for multiple comparisons.

Measures of general and subjective drug effects (VAS, ARCI, LDST scores, and cardiovascular measures) were compared between test sessions via paired-sample *t* tests. One participant was unable to attend for a final test session and so his data were excluded from the analysis. Another participant failed to reach criterion level performance in the acquisition stage of the task on both test sessions, and so his data were also excluded, yielding a final n of 28.

All statistical analyses were carried out in SPSS 19.0 (IBM Corp., Armonk, NY), except the computational modelling analysis, which was implemented in Matlab R2011b (Mathworks, Inc., Sherborn, MA).

## RESULTS

### Baseline-Dependent Effects of Haloperidol on Behavioral Sensation-Seeking

The main findings of Study 1 were replicated in the baseline session data from our second sample of participants (significant relationships in the expected directions between θ values and both individual RT effect and self-reported sensation-seeking) (Supplementary Figure 1). A concordance analysis between data from baseline and placebo sessions also indicated fair-to-good reliability of estimates of θ value across sessions (see Supplementary Information), supporting the validity of our use of a repeated-measures design.

When considering data from the 2 test (drug/placebo) sessions, overall, participants again chose the shock-associated stimulus (CS+) significantly more often on higher points than equal points trials, and on equal compared with lower points trials, on both placebo and drug sessions (main effect of trial type; *F*_2,54_=138.54, *ƞ*_*p*_^*2*^ =0.837, *P* *<* .001; difference between types all *P* *<*.001; mean (± SD) choice on placebo was 0.806±0.19, 0.398±0.17, and 0.126±0.13, respectively, for these trial types, while on haloperidol it was 0.744±0.19, 0.399±0.15, and 0.158±0.15.

There were no significant overall effects of haloperidol treatment on current amplitude, points value assigned to the MES (θ), choice stochasticity (β), mean RT, or relative RT for MES vs non-MES–associated stimuli (all *P*>.1). Drug order (active preparation on first vs second test session) was not a significant between-subjects factor for any of the dependent variables (*P*>.1), and there was no overall drug*drug order interaction (*P*>.1). Therefore, drug order was discarded from the model for subsequent analyses to maximize sensitivity.

**Figure 4.**
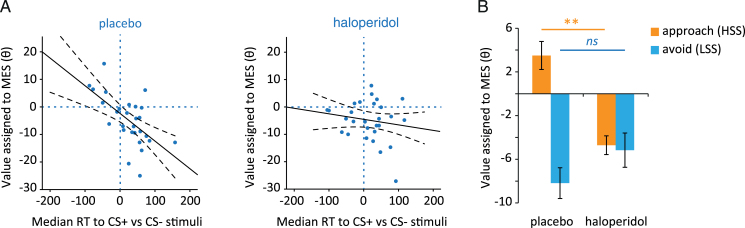
Effects of haloperidol on the value assigned to intense sensory stimulation. (*A*) In a second sample of healthy volunteers, the value assigned to intense sensory stimulation (mild electric stimulation [MES]) was significantly related to relative choice reaction time (RT) for MES vs non-MES–associated stimuli on placebo (*r*=-0.602, *P=*.001), but not under haloperidol (*P*>.1; significant decrease in regression coefficient, *P<*.05). Dashed lines indicate 95% confidence intervals. (*B*) If subjects were divided into those who approached (showed speeded relative RTs towards, n=8) and those who avoided (showed slowed relative RTs towards, n=20) the opportunity for the intense sensory stimulation under placebo, there was a significant interaction between sensation-seeking group and effect of drug (*P<*.01). Haloperidol decreased the economic value assigned to the MES only in those participants who exhibited approach reactions towards MES-associated stimuli under normal conditions (high-sensation seekers [HSS]; *cf* low-sensation seekers [LSS]). Error bars represent SEM. **P=.01, ns *P*>.10, drug vs placebo. n=28.

The strong relationship between the points value participants assigned to receipt of the MES and relative choice RT for MES-associated vs non-MES–associated stimuli observed in Study 1 was replicated in the second sample under placebo conditions (*r*=-0.602, *P=*.001), but, intriguingly, not under haloperidol (*r*=-0.199, *P*>.1) (Figure 4A).

A posthoc analysis revealed that there was indeed a significant attenuation of this relationship under haloperidol (Fisher *r*-to-*Z* transformed Pearson-Filon test for decrease in correlation coefficient; *Z*=-1.735, *P=*.041, 1-tailed; Raghunathan et al., 1996). Thus, haloperidol treatment appeared to abolish the approach-avoidance effect with respect to relative preference for the intense sensory stimulus. Similarly, although self-reported sensation-seeking score was significantly and selectively positively correlated with MES value (θ) on placebo (*r*=0.391, *P=*.040; all other UPPS impulsivity subscale scores unrelated to MES preference, *P*>.1), this was not the case under haloperidol (*r*=-0.127, *P*>.1; Steiger’s *Z* for significant difference in correlation coefficient between drug conditions=2.25, *P=*.024; Steiger, 1980).

Based on the above finding, in conjunction with our previous observation that the effects of a D2-ergic drug may depend on baseline sensation-seeking (Norbury et al., 2013), a further analysis was conducted to check for baseline-dependent drug effects that may have been masked in the group-level analysis. To discover what was driving the attenuation of the RT effect under drug, participants were grouped according to whether they showed conditioned approach (speeded RT to CS+ vs CS- stimuli, ie, individual RT effect <0, N=8) or conditioned suppression (slowed RT to CS+ vs CS- stimuli, ie, individual RT effect >0, n=20) of their responses towards the intense sensory stimulation under placebo conditions.

When this approach or avoid grouping was added to the model as a between-subjects factor, there was a significant interaction between drug treatment and group on value assigned to the MES (significant drug*group interaction on θ value; *F*_1,26_=10.64, *ƞ*_*p*_^*2*^=0.290, *P=*.003; interaction on β value *P*>.1). Simple effects analysis revealed a significant decrease in MES value in the approach group on haloperidol vs placebo (*F*_1,26_=7.97, *ƞ*_*p*_^*2*^ =0.235, *P=*.009). By contrast, there was no effect of drug on MES value in the avoidance group (*P*>.1) (Figure 4B). Thus, haloperidol appeared to selectively attenuate MES value in individuals who exhibited approach behavior towards the intense sensory stimulus under baseline conditions.

Approach and avoid groups did not differ in age, weight, estimated IQ, or self-determined current intensity (independent samples *t* tests, all *P*>.1), but did differ in UPPS sensation-seeking score (*t*_26_=2.261, *P=*.032, significantly higher mean score in the approach group; 40.9±8.1 vs 32.9±8.5). Similarly to Study 1, independent-samples median tests revealed that individuals in the approach group smoked significantly more cigarettes per week than the avoid group (Fisher’s *P=*.022) and showed a nonsignificant trend towards greater weekly alcohol consumption (*P=*.096; mean cigarettes per week 20±25 vs 3.9±13; mean drinks per week 12±13 vs 3.5±3.9).

The effect of haloperidol on θ value (difference in value between drug and placebo sessions) was unrelated to age, weight, estimated IQ, drug effect on overall mood or alertness VAS ratings, drug effect on the sedation or dysphoria scales of the ARCI, or drug effect on general psychomotor function (LDST score; all *P*>.1). There was also no significant relationship between effect of drug on θ value and number of alcoholic drinks consumed or cigarettes smoked in an average week (Spearman’s ρ<0.25, *P*>.1). Subjects who had/had not (n=10 vs n =18) (Table 1) engaged in any recreational drug use other than alcohol or tobacco during the last 12 months did not differ in the effect of haloperidol on θ value (independent samples *t* test, *P*>.1).

### Subjective and General Psychomotor Drug Effects

The above findings could not be explained by generic effects of drug treatment. Overall, there were no significant effects of haloperidol on VAS ratings of mood, affect, or potential physical side effects (16 scales, all *P*>.1) (for details, see Supplementary Table 1). There was also no effect of haloperidol on any ARCI subscale score (MBG euphoria, PCAG sedation, LSD dysphoric and psychotomimetic effects, BG and A stimulant-like effects scales, all *P*>.1) or cardiovascular measures (blood pressure and heart rate, *P*>.1). There was no effect of drug treatment upon participant ratings of whether they believed they were on the drug or placebo session (*P*>.1). Finally, there was no effect of haloperidol on general psychomotor function as indexed by LDST performance (*P*>.1).

### Effects of Drug on Learning

Finally, we examined the hypothesis that the observed effects of haloperidol could be due to differences in learning between drug and placebo sessions. We found no effect of haloperidol on number of trials required to reach criterion performance in the first phase of the task (*P*>.1). Participants’ mean “shock knowledge” ratings for CS+ and CS- stimuli (ratings on a VAS ranging from chance of shock [+300] to no chance of shock [-300]) were entered into a repeated-measures model with the within-subjects factors of drug (haloperidol vs placebo) and CS type (CS+ vs CS-), revealing a significant main effect of CS type (F_1,27_=74.56, *ƞ*_*p*_^*2*^=0.734, *P<*.001; mean [± SEM] rating of CS+ stimuli 146±18.2, mean rating of CS- stimuli -150±19.1), but no effect of drug treatment (*P*>.1) or drug*CS type interaction (*P*>.1) on explicit knowledge of MES associations. When approach vs avoid group was added to the model as a between-subjects factor, there was no difference between groups in the effect of drug on shock knowledge ratings (drug*group, *P*>.1), or the effect of drug depending on CS type (drug*CS type*group, *P=*.09).

## Discussion

We examined how the opportunity to experience an intense sensory stimulus (MES) influenced behavior during a simple economic decision-making task, and, subsequently, how this behavioral index of sensation-seeking was affected by the D2 dopamine receptor antagonist haloperidol. Above chance choice of stimuli associated with intense tactile stimulation occurred reliably in some participants, even when this choice involved the sacrifice of monetary gain. This finding is consistent with the intense sensory stimulation being considered to be appetitive in these individuals. In support of this interpretation, participants who chose a greater proportion of MES-associated stimuli had higher self-reported sensation-seeking scores, increased their “liking” ratings of these stimuli following the introduction of the MESs, and assigned a positive economic value to the opportunity to receive the additional sensory stimulation in a well-fitting computational model of task performance.

Importantly, there was a highly significant relationship between preference for the intense sensory stimulus and choice RTs, consistent with the notion that the MES had motivational significance to participants. In both samples, participants who chose a greater proportion of MES-associated stimuli showed a relative speeding of their responses when choosing these stimuli, with the opposite effect observed in people who tended to avoid them. In conjunction with previous observations that individuals generally show speeded response times for appetitive stimuli but are slower to approach aversive stimuli (Crockett et al., 2009; Wright et al., 2012), this suggests that the opportunity for intense sensory stimulation influenced participants’ choice via an approach-avoidance–like mechanism.

Critically, this effect was not evident under the influence of a D2 receptor antagonist. This was due to a selective decrease in the economic value assigned to receipt of the intense sensory stimulus in participants who exhibited decreased relative RTs towards (or displayed approach reactions to) the MES under placebo conditions (behavioral high-sensation seekers).

The results presented here are in line with a broader background of evidence from both humans and animals that relates trait sensation-seeking to variation in dopaminergic neurotransmission, particularly in striatal regions (Hamidovic et al., 2009; Olsen and Winder, 2009; Shin et al., 2010; Gjedde et al., 2010; Norbury et al., 2013). A combination of evidence from genetic and PET radioligand displacement studies suggests that individuals higher in sensation-seeking personality may have both higher endogenous dopamine levels and greater dopaminergic responses to cues of upcoming reward in the striatum (Riccardi et al., 2006; Gjedde et al., 2010; O’Sullivan et al., 2011). According to one influential model of the role of dopamine in striatal function (Frank, 2005), in the normal state this may contribute to increased inhibition of “NoGo” (action inhibition) pathway neurons via increased stimulation of inhibitory postsynaptic D2 receptors. This in turn would result in greater overall thalamic disinhibition or “Go” bias (favoring action expression) in high-sensation seekers, particularly in the presence of reward cues.

Haloperidol is a silent D2 receptor antagonist (blocks endogenous dopamine signalling via D2 receptors; Cosi et al., 2006), and D2 antagonists have previously been shown to preferentially affect striatal function (Kuroki et al., 1999; Honey et al., 2003). Therefore, it is possible that under haloperidol, the responses of higher sensation seekers may be normalized (increase in resemblance to lower sensation seekers) by allowing increased NoGo pathway output. This would explain our finding of a selective decrease in appetitive reactions to the intense sensory stimulation in the higher sensation-seeking (approach group) individuals.

Our finding of a significant effect of haloperidol on choice, in the absence of any influence on learning, is consistent with recent work suggesting that D2 antagonists may have strong effects on choice of rewarding-predicting stimuli while leaving learning intact (Eisenegger et al., 2014). However, it is important to note that the putative mechanism suggested above assumes a predominantly postsynaptic effect of haloperidol (Frank and O’Reilly, 2006). Despite our attempt to ensure significant postsynaptic receptor binding by use of a greater dose than the previously cited study (where mixed pre- and postsynaptic D2-ergic effects were thought to be observed), we can provide no direct evidence of this. Further, inferences regarding the brain regions involved in our findings are speculative and would need to be tested in further work, for example involving functional imaging.

The studies presented here have some limitations. First, as sensation-seeking behaviors in the real world can take many different forms, it might appear surprising that use of a single, tactile sensory stimulus (MES) is able to sufficiently capture sensation-seeking behavior in all individuals. However, our findings are consistent with a previous study reporting distinct physiological response profiles to electric shock in low and high self-reported sensation-seekers (De Pascalis et al., 2007). We would not seek to claim that performance on our task captures all of sensation-seeking personality, as this is a complex multidimensional trait, but it may tap operational sensation-seeking–like behavior in at least a subset of high-sensation–seeking individuals, thereby allowing us to probe underlying neural mechanisms in the laboratory (eg, with pharmacological manipulations). In analogous fashion, there is some evidence that apparently dissimilar animal operationalizations of sensation-seeking behavior may tap at least partially overlapping neural circuitry (eg, Parkitna et al., 2013).

Crucially, in both our studies, choice of MES-associated stimuli was found to correlate selectively with total self-reported sensation-seeking scores, which probe multiple classes of sensation-seeking–type behaviors. Although these relationships were of only moderate strength, it should be noted that these findings are at the higher end of the range of those generally found between behavioral and questionnaire measures of impulsive behavior (Helmers et al., 1995; Mitchell, 1999). We also found some evidence of greater recreational substance consumption amongst individuals who assigned a positive value towards opportunity to experience the MES, indicating that task performance may relate to real-life engagement in sensation-seeking behaviors.

Second, as our drug finding is based on a significant decrease in value in one (previously higher mean value) subgroup, an alternative explanation of our findings from Study 2 is that this simply represents a regression to the mean effect. However, against this interpretation, we found evidence of fair-to-good reliability of θ values generated from the same participants across multiple sessions of our novel paradigm (Supplementary Information).

Furthermore, the subgrouping for Study 2 is based on individual difference in relative choice RTs rather than θ values per se (although the 2 are significantly correlated). We also used our estimate of RT effect from the second or third testing session (placebo session) to group participants, a strategy that has previously been argued to help guard against regression to the mean effects (Barnett et al., 2005). Taken together, we would contend that these factors argue against a purely trivial effect of haloperidol on MES value in the approach or high-sensation–seeking individuals.

Third, although haloperidol is considered to be a selective D2 receptor antagonist (it binds >15 times more strongly to D2 than D1 receptors in rat and human cloned cells; Arnt and Skarsfeldt, 1998), it has also been shown to have modest affinity for the *α*-1 adrenoreceptor and the serotonin 2A receptor in postmortem human brains (Richelson and Souder, 2000). Therefore, we cannot be completely certain about the mechanism underlying our drug effects. As haloperidol has previously been reported to induce high levels of brain D2 receptor occupancy at relatively low oral doses (60–70% at 3mg and 53–74% at 2mg; Nordström et al., 1992; Kapur et al., 1997), we are confident that the dose used in our study (2.5mg) was sufficient to antagonize central D2 receptors in our participants. Another potential limitation is the possibility that the behavioral effects we observed are due to some general effect of haloperidol treatment, for example, increased negative affect in some participants. However, the effect of drug on MES value was unrelated to differences in mood, affect, sedation or dysphoria ratings, or our measure of general psychomotor function between drug and placebo sessions.

In summary, the novel paradigm introduced here appears to tap a dimension of willingness to self-administer intense and unusual sensory stimulation, together with associated behavioral invigoration. For participants who choose to approach rather than avoid this kind of stimulation, we propose that it is intrinsically rewarding and that, similar to analogous findings from the animal literature, this appetitive response involves the D2 receptor dopamine system. These findings may aid investigation of various psychopathologies for which more extreme sensation-seeking scores constitute a vulnerability factor.

## Interest Statement

J.P.R. is a consultant for Cambridge Cognition and has participated as a paid speaker in a media advisory board for Lundbeck. All other authors have no financial interests to disclose.
